# Genomic Insights into the Population Genetics and Adaptive Evolution of Yellow Seabream (*Acanthopagrus latus*) with Whole-Genome Resequencing

**DOI:** 10.3390/ani15050745

**Published:** 2025-03-05

**Authors:** Yuan Li, Jingyu Yang, Yan Fang, Ran Zhang, Zizi Cai, Binbin Shan, Xing Miao, Longshan Lin, Puqing Song, Jing Zhang

**Affiliations:** 1 Third Institute of Oceanography, Ministry of Natural Resources, Xiamen 361005, China; liyuan@tio.org.cn (Y.L.); yangjingyu@tio.org.cn (J.Y.); 18110677050@163.com (Y.F.); iamzhangran@163.com (R.Z.); caizizi@mails.ccnu.edu.cn (Z.C.); miaoxing@tio.org.cn (X.M.); linlsh@tio.org.cn (L.L.); 2 Fujian Provincial Key Laboratory of Marine Fishery Resources and Eco-Environment, Xiamen 361021, China; 3 Fisheries College, Jimei University, Xiamen 361021, China; 4 School of Life Science, Central China Normal University, Wuhan 430079, China; 5 South China Sea Fisheries Research Institute, Chinese Academy of Fisheries Sciences, Guangzhou 510300, China; shanbinbin@yeah.net

**Keywords:** *Acanthopagrus latus*, whole-genome resequencing, genetic structure, population history dynamics, adaptive evolution

## Abstract

Yellow seabream (*Acanthopagrus latus*), a species of significant economic importance, predominantly inhabits the warm waters of the Indo-Western Pacific. While previous studies have explored the genetic diversity of *A. latus* using microsatellites and other nuclear markers, a comprehensive understanding of its genetic characteristics and adaptive evolution using whole-genome resequencing (WGR) remains limited. In this study, we collected 60 individuals from six distinct geographic locations and performed WGR, achieving an average sequencing depth of 12.59×, which resulted in the identification of 19,488,059 high-quality single-nucleotide polymorphisms (SNPs). The nucleotide polymorphism (π_θ_) across all populations was consistent, ranging from 0.003042 to 0.003155, indicating low genetic differentiation among populations. Comparative analyses revealed that populations other than that in Xiamen (XM) have undergone adaptive evolution, potentially linked to traits such as growth and development, feeding, immunity, and movement. This study explores the population genetics and adaptive evolutionary patterns of *Acanthopagrus latus* at the genomic level, providing an essential foundation for the conservation and management of this economically important species in the future.

## Simple Summary

Yellow seabream (*Acanthopagrus latus*) is an economically significant fish species in the Indo-Western Pacific region, yet its genetic characteristics and adaptive evolution remain insufficiently studied. In this study, we conducted whole-genome resequencing on 60 individuals from six geographic regions, identifying nearly 19.5 million genetic variations. The results showed low genetic diversity among populations. Additionally, compared to the Xiamen population, which is subject to zero fishing pressure, populations from the other five regions exhibited genetic changes associated with traits such as growth, feeding, immunity, and movement. This study systematically reveals the genetic and evolutionary patterns of *A. latus*, providing crucial scientific insights into the conservation of this important economic species and its sustainable management under changing environmental conditions.

## 1. Introduction

Yellow seabream (*Acanthopagrus latus*), which belongs to Actinopterygii, Perciformes, Sparidae, is primarily distributed in the warm waters of the Indo-Western Pacific. This species inhabits shallow coastal waters and estuaries and does not typically engage in long-distance migration [1]. Despite its economic importance, natural populations of *A. latus* have declined due to intense fishing. As a result, it has become a significant aquaculture species, with stock enhancement efforts underway in order to restore its natural population [2]. In recent years, there has been increasing interest in the genetic background of this species and in the conservation and utilization of resources [3,4,5,6].

With the discovery of genomes for an increasing number of species, whole-genome resequencing technology has developed rapidly [7]. By using the known genome sequences as references, we can obtain variation information for individuals or populations, such as SNP, InDel, SV, and CNV [8]. This approach is applicable in many fields, including population genetics and evolutionary analysis [9,10]. Extensive variation loci can reveal the genetic characteristics of populations and identify candidate genes for selection [11]. For instance, a complete genetic break between the Chinese and Japanese groups of *Sillago japonica* was detected using population genomics based on whole-genome resequencing, indicating that parallel evolution at the genetic level may have been induced by similar temperature regimes in these isolated populations [12]. Moreover, whole-genome resequencing, combined with geological events, can be used to perform historical dynamics analysis and reveal evolutionary processes [11].

Research on adaptive evolution in the environment has attracted increasing attention in recent years [13]. For example, adaptive evolution in euryhaline bivalves involves osmoregulation, the scavenging of reactive oxygen species (ROS), energy metabolism, and adjustments to membrane lipids [14]. As the intensity of fishing has increased, fish have gradually undergone adaptive evolution in order to cope with the pressure of fishing and continue reproducing, which is known as fishing-induced evolution (FIE) [15]. FIE, caused by size-selective harvesting, alters the life history traits of fish, leading to earlier sexual maturation and a reduction in the body size of adults [16]. Historically, FIE research has relied on experimental ecological methods due to the limited field data available, using genetic markers to compare different individuals within a species and explore the impact of fishing on genetic composition. With the advancement of next-generation sequencing technology, genomic methods could be used to identify selection-related loci, making them a powerful tool in future FIE research [15].

Currently, research on the genetics of *A. latus* mainly focuses on areas such as systematics and genetic structure. For example, Peng et al. [17] were the first to isolate and identify a large number of genome-wide microsatellite markers from the genome of the yellowfin seabream. They determined the locations of these microsatellites in exons, introns, and intergenic regions, providing a theoretical basis for studying the effects of microsatellites on gene function. Liu et al. [4] used mitochondrial DNA control region (CR) markers to analyze the genetic diversity and structure of yellowfin seabream populations from three sites in Dongshan Bay and one site in Xiamen, evaluating the current status of their genetic resources. In 2022, a significant breakthrough was made in chromosome-level genome research on *A. latus*, in which PacBio and Hi-C sequencing strategies were combined to achieve the chromosome-level assembly of the yellowfin seabream genome; this opened new avenues for future research [18]. In genomic research, Wang et al. [19] used whole-genome resequencing and genotype–environment association analysis to provide insights into the genetic description and adaptive potential associated with salinity-induced evolutionary changes.

The Xiamen Rare Marine Species National Nature Reserve, established in 2000, is located in Xiamen Bay and its surrounding waters, covering several important ecological areas. The core protected area covers 75.88 square kilometers and is home to rich marine biodiversity, with rare species such as the *Sousa chinensis* and the *Bratfchiostoma belcheri* [20]. It is one of China’s key marine ecological reserves. The reserve was established to restore and maintain the habitats of these rare species through strict protection measures and to reduce the ecological pressure caused by human activities. In recent years, with continuous protection efforts, the ecological environment in the reserve has gradually improved, biodiversity has been effectively restored, and the population numbers and health of key species have increased. In particular, the recovery of species resources has been significant in areas with lower fishing pressure [21].

Xiamen Bay, as an important part of this reserve, with fishing restrictions and ecological restoration measures in place, has become an ideal experimental site for studying marine ecological changes and the impact of fishing pressure [22]. In this study, we used whole-genome resequencing technology to reveal the genetic structure of the *A. latus* population and used Xiamen Bay as a control group to explore the impact of different fishing pressures on the environmental adaptability and evolution of *A. latus*. The findings of this study will provide new insights for the conservation of *A. latus*.

## 2. Materials and Methods

### 2.1. Sample Collection and DNA Extraction

**Figure 1:**
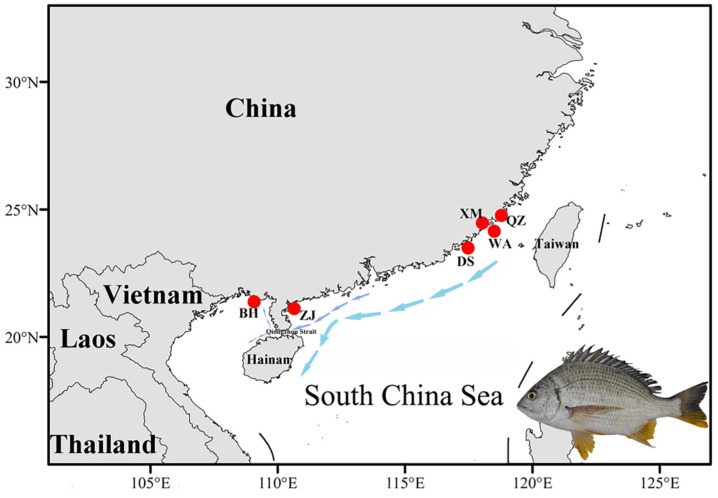
Sampling sites of *A. latus* (the red dots indicate the sampling locations, and the arrows represent the coastal currents along the South China coast during autumn and winter. A morphological diagram of *A. latus* is shown in the lower right corner).

The samples were collected along the southeastern coast of China from September to October 2022, with specific sampling locations including the coastal waters of Beihai (BH) in Guangxi, Zhanjiang (ZJ) in Guangdong Province, and Xiamen (XM), Quanzhou (QZ), Dongshan (DS), and Wuyu (WA) in Fujian Province. Ten individuals were collected from each location, with detailed information provided in Figure 1 and Table 1. All samples were identified based on their morphological characteristics [23]. Muscle tissue samples were taken from each individual and stored in 95% ethanol or frozen for DNA extraction. A total of 60 individuals (10 samples from each population) were used for whole-genome resequencing. Genomic DNA was extracted using the TIANamp Marine Animals DNA Kit (Tiangen Biotech (Beijing) Co., Ltd., Beijing, China). To assess the quality of the extracted DNA, the concentration and purity of the DNA samples were measured using a NanoDrop 2000 spectrophotometer (Thermo Fisher Scientific Inc., Waltham, MA, USA). The integrity of the genomic DNA was evaluated using agarose gel electrophoresis. The specific submission criteria were as follows: DNA concentration ≥ 100 ng/μL, total amount ≥ 50 μg, and an A260/A280 ratio between 1.8 and 2.0. Electrophoresis showed no significant RNA bands, with clear and intact genomic bands; in addition, the major band was expected to be larger than 100 kb.

### 2.2. Genomic Library Construction and Sequencing

Genomic libraries were constructed by randomly interrupting the DNA using an ultrasonic crusher; this was followed by end repair, adapter ligation, and PCR amplification. The DNA concentration was determined using the Qubit 2.0 spectrophotometer (Thermo Scientific, Waltham, MA, USA), and the library integrity was assessed using the Agilent Bioanalyzer 2100 system (Agilent Technologies, Santa Clara, CA, USA). Sequencing was performed on an Illumina platform (Illumina, San Diego, CA, USA) using a 150 bp paired-end strategy at Personalbio Technology Co., Ltd. (Qingdao, China).

### 2.3. Sequencing Data Quality Control

Raw data were filtered and quality metrics were computed using Fastp software (v0.23.4) [24]. The filtering process included the following steps: (1) the removal of reads containing adapter contamination, (2) the establishment of a window size of 5 bp and a step size of 1 bp for quality filtering, retaining bases in windows with an average Q value > 20, and (3) the removal of paired-end reads with a length ≤ 50 bp.

### 2.4. Sequence Alignment

The reference genome of the second and third-generation mixed xanthopterus was used as the sequence (GCA_904848185.1) [18], and the sequences were compared using BWA software (0.7.11). The results were converted to a bam file using samtools (v0.7.17-r1188) [25] and sorted using Picard (v1.117) software. Reads that exhibited map Q ≥ 20 and were properly paired were used to find variations. GATK (v4.2.0) software was used to perform sequence realignment and correction near InDel to improve the accuracy of the SNPs [26].

### 2.5. Variant Calling and Annotation

GATK (v4.2.0) was used to call variation in the SNPs and InDels [26]. HaplotypeCaller was used to generate gVCF files from each BAM file, which were then merged using Combine GVCFs. Variants were discovered with Genotype GVCFs, and SNPs and InDels were filtered using VariantFiltration and the following parameters: for SNPs, QD < 2.0||MQ < 40.0||FS > 60.0||SOR > 3.0||MQRankSum < −12.5||ReadPosRankSum < −8.0; for InDels, QD < 2.0||FS > 200.0||SOR > 10.0||MQRankSum < −12.5||ReadPosRankSum < −8.0. Structural variants (SVs) and copy number variations (CNVs) were detected using Lumpy (v0.2.13) [27] and CNVnator (v0.4.1) software [28], respectively. For Lumpy, the parameter -mw (minimum weight across all samples for a call) was set to improve the quality control of SV detection, while for CNVnator, the key parameter bin_size was set to 200. ANNOVARR (https://annovar.openbioinformatics.org/en/latest/ (accessed on 15 June 2024)) [29] was used to annotate the genomic locations and functional effects of variants. SNPs were classified according to their chromosomal positions (such as intergenic, exonic, intronic, splicing regions, and 1 kb upstream and downstream regions) and their functional effects (including synonymous, nonsynonymous, and stop codon gain or loss). Variants were filtered based on a minor allele frequency > 0.01, a depth > 3, and a missing rate < 0.2.

### 2.6. Population Genetic Structure Analysis

All SNPs were used to construct a phylogenetic tree based on the neighbor-joining method using the default parameters of PHYLIP (v3.697) software. The PLINK (v2.0) software [30] was used to filter the linkage SNP, convert the VCF file to a PHYLIP file, and construct the phylogenetic tree. MEGA (v5.0) [31] was used for visualization. The genetic structure of the SNP data was analyzed using ADMIXTURE software (v1.3.0) [32]. ADMIXTURE was implemented using default settings to test the statistical likelihood of one to six populations. First, the VCF file was converted into the input format for ADMIXTURE using PLINK (v2.0) software [30]. Then, analysis was performed for K values ranging from one to six, and the cross-validation (CV) errors for all results were compared. To make the calculation more accurate, each K value was repeated 20 times [33], and the corresponding CV values were recorded. Finally, the result with the minimum CV for each K was selected for presentation.

### 2.7. Population Genetic Diversity and Genetic Differentiation Analysis

The VCFtools software (v0.1.16) [34] was used to segment the data with a 10 kb step and 100 kb window (− window-pi 100,000 − window-pi-step 10,000); it was also used to perform pairwise calculations to obtain the Fixation Index (*F_st_*) values and total nucleotide diversity (π_θ_) values for each population.

### 2.8. Gene Flow Analysis

Gene flow analysis was conducted using Treemix (v1.13) software [35] to visualize the inter-population gene flow among the 60 *A. latus* samples. To account for linkage disequilibrium, a window size of 1000 was applied (−k), and the “−global” option was selected to generate the ML tree. Migration events (−m 0 1 2 3 4 5) were progressively incorporated into the tree to assess different gene flow scenarios.

### 2.9. Effective Population Size Analysis of Evolutionary History

In this study, the sample with the highest sequencing depth was taken from each *A. latus* group, and the pairwise sequentially Markovian coalescent (PSMC) [36] was used to determine the historical effective population size for the six groups. The PSMC (https://github.com/lh3/psmc (accessed on 15 June 2024)) software’s “fq2psmcfa” and “splitfa” tools were used to generate the input file for modeling. The PSMC command included the following parameters: “−N25” for the number of algorithm cycles, “−t15” for the upper limit of the most recent common ancestor (TMRCA), “−r5” for the initial θ/ρ, and “−p 4 + 25 × 2 + 4 + 6” for the atomic intervals. The population history was plotted using the “psmc_plot.pl” script. The point mutation rate was set to 2.5 × 10^−8^, and the generation time was set to 1 year [12].

### 2.10. Detection of Selective Sweeps

Selective sweep analyses were performed using the Xiamen *A. latus* population (zero control point for fishing pressure) as a reference. The reduction in diversity (ROD) between populations was calculated based on the nucleotide diversity (π_θ_), with ROD = |1 − π_θ_/π_θ_XM|. The top 5% values for both *F_st_* and ROD were used to identify selected genomic regions. The functional annotation and enrichment analysis of candidate genes were performed by annotating genes with strong selection signals in each population using the eggNOG-mapper (https://github.com/jhcepas/eggnog-mapper/wiki (accessed on 15 June 2024)) software [37], and by performing Gene Ontology (GO) term classification and Kyoto Encyclopedia of Genes and Genomes (KEGG) pathway enrichment analysis using the OmicShare tools (http://www.omicshare.com/tools (accessed on 15 June 2024)) [38,39].

## 3. Results

### 3.1. Data Quality Control

A total of 522.97 Gb of raw data were obtained for 60 *A. latus* samples. The average data size for each sample were 8.72 Gb, with an average sequencing depth of 12.59×, an average GC content of 42.82%, an average Q20 of 97.24%, and an average Q30 of 92.85%. After filtering, approximately 509.20 Gb of clean data were obtained.

### 3.2. Variation Loci Distribution and Genetic Diversity

**Figure 2:**
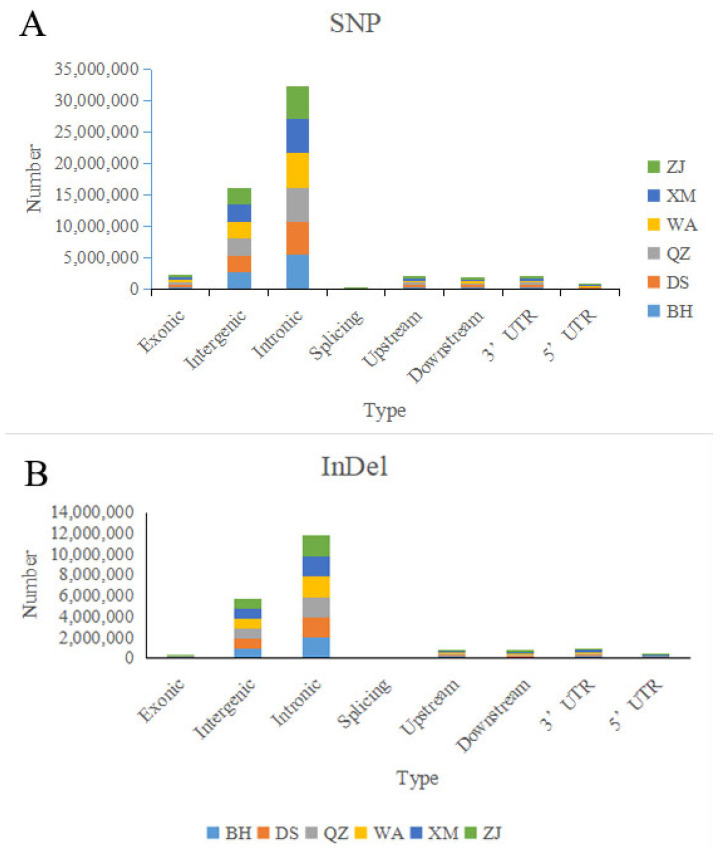
The distribution of SNPs (**A**) and InDels (**B**).

By mapping the reference genome, we identified 19,488,059 SNP loci, 6,515,512 InDel loci, 78,387 SV loci, and 9249 CNV loci across the 60 *A. latus* individuals. SNPs were most numerous in intronic regions (10,965,511), followed by intergenic (5,460,423), upstream (661,882), downstream (623,660), exonic (790,510), and splicing (2655) regions (Figure 2A). Within the gene coding region, 457,790 SNPs were identified as causing synonymous mutations. InDels were most numerous in intronic regions (3,745,973), followed by intergenic (1,816,535), upstream (271,305), downstream (223,498), exonic (48,254), and splicing (3146) regions (Figure 2B). The number of SVs was highest in intronic regions (41,200), while CNVs were most numerous in exonic regions (5539).

The nucleotide diversity (π_θ_) was calculated based on all SNP loci, ranging from 3.042 × 10^−3^ (DS) to 3.155 × 10^−3^ (BH), indicating comparable diversity among the six populations (Table 2).

### 3.3. Population Genetic Structure and Gene Flow

**Figure 3:**
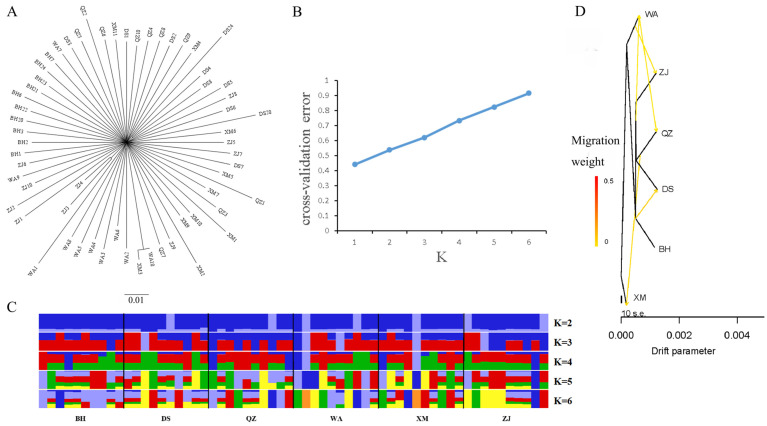
The phylogenetic relationship of six *A. latus* populations. (**A**) The NJ tree of six *A. latus* populations based on all SNPs. (**B**) Cross-validation (CV) error for varying values of K. (**C**) Population genetic structure of *A. latus*. The length of each color fragment indicates the proportion of individual genes inferred from the ancestral population (K = 2~6), and sample names are at the bottom. Each color represents a different hypothetical ancestor. (**D**) Gene flow of *A. latus* among the six populations. The five yellow arrows correspond to the five gene flow events identified in the analysis.

The NJ tree constructed by all SNP sites showed that no obvious genealogical branch corresponding to the sampling sites was detected for *A. latus* in this study (Figure 3A). We calculated the cross-validation error (CV error) at different K values, and the results showed that when K = 1, the error rate was the lowest (Figure 3B). When the number of ancestor groups increased, the *A. latus* populations were further subdivided; however, there was no significant difference among populations (Figure 3C). Furthermore, the pairwise *F_st_* for the six populations ranged from −9.355 × 10^−4^ to −1.016 × 10^−3^ (Table 3). All the *F_st_* values were negative, indicating that genetic differentiation mainly existed within populations.

The gene flow among six populations was evaluated using Treemix. The gene exchanges among populations were evaluated if they fit the maximum likelihood model. There are five gene flows between populations according to the results. This is shown in Figure 3D.

### 3.4. Population Historical Dynamics

**Figure 4:**
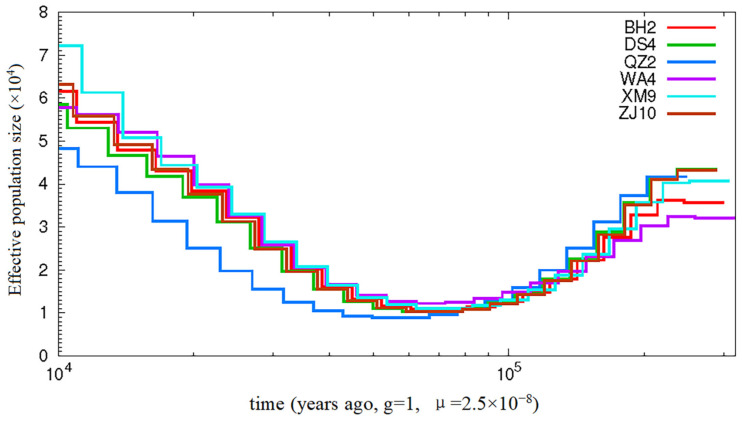
Demographic history of *A. latus*.

The historical effective population (Ne) size was estimated using the PSMC method, and the mutation rate of *A. latus* was set to 2.5 × 10^−8^. One individual from each population was randomly chosen, and the change trends of all six specimens remained consistent. The mutation rate of *A. latus* was set to 2.5 × 10^−8^, and the generation time was set to 1 year. As shown in Figure 4, *A. latus* populations experienced a severe population decline, with relatively small Ne values around 50,000 to 80,000 years ago.

### 3.5. Screening and Enrichment Analysis of Selected Genes

**Figure 5:**
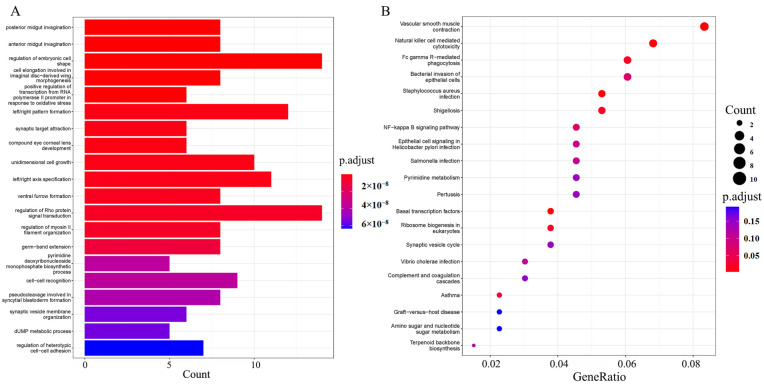
GO (**A**) and KEGG (**B**) enrichment analyses for selected genes in Xiamen and five other locations with *A. latus* populations.

**Figure 6:**
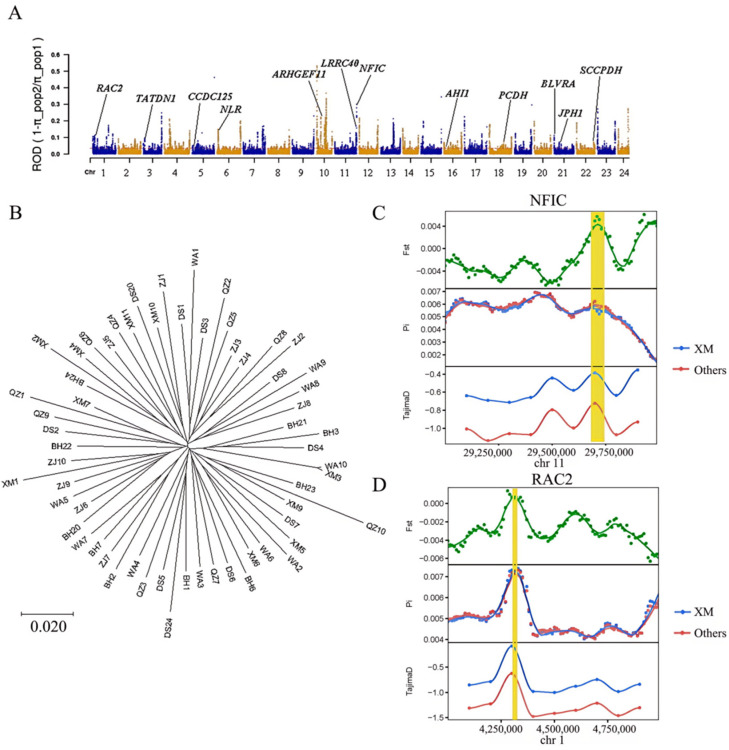
The identification of selection sweeps between Xiamen (XM) and five other locations for *A. latus*. (**A**) ROD values along chromosomes (the yellow/blue dots represents the ROD value of all SNPs, and the red dashed line represents the threshold line of the top 5% of ROD). (**B**) The NJ tree of selected genes. (**C**) *F_st_*, π_θ_, and Tajima’s D near the *NFIC* gene. (**D**) *F_st_*, π_θ_, and Tajima’s D near the *RAC2* gene. The yellow highlight indicates gene regions with strong selective signals. The green wavy line represents the genetic differentiation analysis between the Xiamen population and other populations.

The selected gene regions of five *A. latus* populations were identified, and candidate genes were screened via GO and KEGG enrichment analyses, using population XM as the control population. The GO analysis showed that the GO terms were mainly enriched in the regulation of embryonic cell shape, the regulation of Rho protein signal transduction, left/right pattern formation, left/right axis specification, and unidimensional cell growth. The results of the KEGG analysis revealed that candidate genes were mainly enriched in natural-killer-cell-mediated cytotoxicity, vascular smooth muscle contraction, Fc gamma R-mediated phagocytosis, and the bacterial invasion of epithelial cells (Figure 5). Compared to the control group, NFIC, RAC2, NLR, AHI1, TATDN1, CCDC125, SCCPDH, LRRC40, BLVRA, PCDH, ARHGEF11 and JPH1 may be the candidate genes related to environmental adaptive evolution (Figure 6).

## 4. Discussion

As a valuable and popular commercial fish in China and the Indo-Western Pacific Ocean, *A. latus* has garnered significant research interest regarding its genetic status. Previous studies predominantly utilized mitochondrial DNA fragments as molecular markers [5,40]. However, relying on mitochondrial DNA is unwise due to its relatively narrow genetic scope and low coverage. Although some microsatellite loci have been developed, there has been a lack of population genetic research based on nuclear gene markers. In 2022, the publication of the chromosome-level genome of *A. latus* marked a significant advancement, opening new avenues for research [18]. Whole-genome resequencing technologies have been employed in studies focusing on the assignment of parentage, local adaptation, and population structure [19,41]. Although a large number of SNPs were obtained in this study, there were no significant differences in the genetic diversity parameter π_θ_ between Xiamen Bay and other populations, indicating that there were high levels of homogeneity among the populations. This genetic homogeneity may be attributed to the relatively few geographical barriers between the studied regions, which facilitates gene flow and reduces genetic differentiation. Further analysis supports this conclusion. The NJ tree constructed using all SNP sites did not reveal any clear genealogical branches corresponding to the sampling locations (Figure 3A). Additionally, we calculated the CV error at different K values, and the results showed that the error rate was lowest when K = 1 (Figure 3B). As the number of ancestral groups increased, the *A. latus* populations were further subdivided, but no significant differences were observed among the populations (Figure 3C). These results suggest that, although there is some genetic variation among the samples from different sampling locations, the genetic structure across populations is generally consistent, with low genetic differentiation.

Two genetic management units, divided by the Qiongzhou Strait, have been identified in *A. latus* using the mitochondrial DNA control region and Cyt b sequences [40,42]. Wang et al. [19] suggested that the genetic patterns of this population are influenced by various factors, including salinity gradients, the habitat distance, and ocean currents. The genotype–environment association analysis provided insights into the delineation and adaptive potential associated with salinity-induced evolutionary changes from a whole-genome resequencing perspective. However, no genetic differentiation was detected for the six populations assessed in the present study. All *F_st_* values were negative, indicating that genetic differentiation mainly existed within populations. Frequent gene exchange may be the main reason for this high genetic homogeneity. Although the Qiongzhou Strait may be an important physical barrier for some marine fish species [43], the recent artificial stock enhancement of *A. latus* could strengthen this gene exchange by releasing some non-native individuals. For example, we found high levels of gene flow from population BH to population DS. In addition, the larval stage of *A. latus* is more than 30 days [44], and the ability of its eggs and larvae to disperse via ocean currents may help maintain gene exchange among populations.

Marine ecosystems are currently suffering from severe over-fishing and pollution. The establishment of Marine Protected Areas (MPAs) plays an important role in the prevention of over-exploitation and the protection of fishery resources, thus improving the sustainability of fisheries [45]. Previous studies have shown that fishing pressure may lead to environmental adaptive evolution in fish growth, feeding, immunity, which is known as fishing-induced evolution (HIE) [15]. The adaptive evolution caused by different fishing pressures may occur at any time, but we still know little about its genomic basis. Size-selective harvesting can lead to various phenotypic changes, such as those in the growth rate of fish [46]. Moreover, these fishery-induced phenotypic changes have been found to be partly genetically determined [46]. In MPAs, fishing activities are strictly prohibited, effectively reducing the fishing pressure to zero. This provides an ideal environment for studying the natural genetic adaptations of marine species without the interference of fishing pressure. By comparing populations within MPAs (XM) with those under normal fishing pressure (BH, ZJ, DS, WA, QZ), researchers can determine the impact of varying levels of human activity on the genomic structure and ecological adaptability of the target species. These comparative studies help uncover the genetic basis of marine organisms’ responses to environmental changes and fishing pressure, offering crucial insights into their adaptability and resilience. Such research not only deepens our understanding of marine biodiversity but also provides valuable data for the development of effective conservation and management strategies, helping to protect these species in the face of increasingly severe environmental challenges.

In the present study, the selective sweep analyses and gene annotation indicated that NFIC, RAC2, NLR, AHI1, TATDN1, CCDC125, SCCPDH, LRRC40, BLVRA, PCDH, ARHGEF11, and JPH1 may be the candidate genes associated with environmental adaptive evolution for the other five populations. The nuclear factor one (NFI) family usually encodes four site-specific transcription factors, namely NFIA, NFIB, NFIC, and NFIX [47]. For mammals, NFIC is expressed in many organ systems, but Steele-Perkins et al. found that defects in the development of the tooth root were the prominent phenotype [48]. In this study, it was found that *NFIC* genes may play an important role in the development of the teeth, bones, and scales of *A. latus* in areas with high fishing pressure. As an important member of the Ras gene family, Ras-related C3 botulinum toxin substrate protein (RAC2) exerts a variety of biological functions; these functions are closely connected with the formation of the cytoskeleton, cell signal transduction, cell adhesion, and cell apoptosis [49]. In an experiment on the resistance of the northern snakehead (*Channa argus*) to Aeromonas veronii infection, the *RAC2* gene was inferred to play an important role in the immune response of *C. argus* [49]. In the present study, compared with the Xiamen population, the *RAC2* gene was found to be particularly important in the immune response of *A. latus* to pathogens, the regulation of cell signaling, the formation of the actin cytoskeleton, and the survival of hematopoietic cells in areas with high levels of fishing pressure.

NLR receptors have been found to potentially participate in the recognition of intracellular pathogenic microorganisms and inflammatory response processes [50]. The AHI1 gene is thought to play an important role in the formation and maintenance of the outer segment of photoreceptors, as well as in the morphogenesis of the distal pronephric duct cilia of zebrafish [51]. TATD is a DNase domain-containing protein, which is a conserved gene found in various organisms, including archaebacteria and eukaryotes. For zebrafish, the *TATDN1* gene plays an important role in chromosome segregation and eye development [52]. The *CCDC125* gene is involved in the negative regulation of GTP enzyme activity, cell motility, and Rho protein signal transduction [53]. The *SCCPDH* gene can activate oxidoreductase activity and participate in the biosynthesis of glucose and lipids [54]. The *BLVRA* gene can eliminate dangerous reactive oxygen species, making it particularly important for cell health [55]. The *ARHGEF11* gene is involved in regulating cell growth, cell motility, Rho protein signaling, and cytokinesis [56]. The *JPH1* gene plays an important role in the muscles and heart of fish [57]. The *RAC2* and *NLR* genes are thought to be involved in the immune response of *A. latus* in populations with high fishing pressure. A large number of immune-related pathways and functional enrichment have been found in the enrichment analysis of selective clearance genes, which may be related to the strong resistance of *A. latus* to diseases under high fishing pressure. The selection of *NFIC*, *TATDN1*, and *AHI1* genes may affect their feeding and exercise abilities, while the *CCDC125*, *SCCPDH*, *BLVRA*, *ARHGEF11*, and *JPH1* genes may promote the growth and metabolism of the cells and tissues of *A. latus*.

## 5. Conclusions

In conclusion, this study obtained and annotated a large volume of information regarding SNP, InDel, CNV, and SV variations from six geographic populations of *Acanthopagrus latus*; this enabled a genomic variation database to be constructed for the species. The phylogenetic tree and population structure analyses indicated that there was no clear distinction among the different geographic populations of *A. latus*, with weak geographic separation and no significant genetic differentiation observed. The differences in genetic diversity among populations were also minimal. Additionally, in regions with high fishing pressure, *A. latus* may have undergone environmental adaptive evolution with regard to growth, development, feeding, immunity, and movement.

## Figures and Tables

**Table 1:** Sampling locations and sample size of *A. latus*.

Sampling Sites	Sampling Coordinates	Number of Samples
Beihai (BH)	21°27′23.2″ N; 108°51′59.3″ E	10
Zhanjiang (ZJ)	21°09′35.5″ N; 110°44′02.1″ E	10
Dongshan (DS)	23°37′48.1″ N; 117°21′01.2″ E	10
Wuyu (WA)	24°20′11.7″ N; 118°08′22.5″ E	10
Xiamen (XM)	24°25′35.5″ N; 118°04′49.9″ E	10
Quanzhou (QZ)	24°49′41.8″ N; 118°43′56.5″ E	10

**Table 2:** Nucleotide polymorphism (π_θ_) of all populations.

	Beihai	Dongshan	Quanzhou	Wuyu	Xiamen	Zhanjiang
π_θ_	3.155 × 10^−3^	3.042 × 10^−3^	3.058 × 10^−3^	3.119 × 10^−3^	3.096 × 10^−3^	3.102 × 10^−3^

**Table 3:** Matrix of pairwise *F_st_* values between six *A. latus* populations based on all SNPs.

	Beihai (BH)	Dongshan (DS)	Quanzhou (QZ)	Wuyu (WA)	Zhanjiang (ZJ)	Xiamen (XM)
BH						
DS	−7.971 × 10^−4^					
QZ	−9.355 × 10^−4^	−1.572 × 10^−3^				
WA	−8.193 × 10^−4^	−1.044 × 10^−3^	−1.336 × 10^−3^			
ZJ	−4.121 × 10^−4^	−8.160 × 10^−4^	−1.016 × 10^−3^	−7.174 × 10^−4^		
XM	−1.016 × 10^−3^	−1.420 × 10^−3^	−1.670 × 10^−3^	−4.395 × 10^−3^	−1.034 × 10^−3^	

## Data Availability

The datasets used in this study are available in online repositories. The study data have been deposited in the NCBI repository under the BioProject accession number: PRJNA1149977 and PRJNA1141615 (SAMN42898516-SAMN42898525).

## References

[1] Iwatsuki Y. (2013). Review of the *Acanthopagrus latus* complex (Perciformes: Sparidae) with descriptions of three new species from the Indo-West Pacific Ocean. J. Fish Biol..

[2] Lv S., Wang X., Li C. (2019). Optimization of key procedures for fish tagging and releasing with its application to yellowfin seabream (*Acanthopagrus latus*). J. Fish. China.

[3] Ye Y., Huang Z., Xu A., Wang J., Li Z. (2022). Genetic diversity and genetic structure of *Acanthopagrus latus* populations in the southeastern coast of China based on the mitochondrial DNA control region analysis. J. Appl. Oceanogr..

[4] Liu C., Zhang J., Liu S., Song P., Guan Y., Shan B., Li Y., Lin L. (2021). Genetic diversity of the yellowfin seabream, *Acanthopagrus latus* (Actinopterygii: Perciformes: Sparidae)-an enhancement species in Dongshan Bay. Acta Ichthyol. Pisc..

[5] Song P., Liu C., Cai Z., Liu S., Xiang J., Shan B., Ji D., Li Y., Lin L. (2021). Genetic characteristics of yellow seabream *Acanthopagrus latus* (Houttuyn, 1782) (Teleostei: Sparidae) after stock enhancement in southeastern China coastal waters. Reg. Stud. Mar. Sci..

[6] He Z., Xiao S., Wang S.S., Chen H.-F., Su S., Zhao Y., Yang C., Zeng D., Zhu W., Chen X. (2021). Genetic structure of D-loop sequence in *Acanthopagrus latus*. J. Fish. China.

[7] North H.L., McGaughran A., Jiggins C.D. (2021). Insights into invasive species from whole-genome resequencing. Mol. Ecol..

[8] Wang J., Hu Z., Liao X., Wang Z., Li W., Zhang P., Cheng H., Wang Q., Bhat J.A., Wang H. (2022). Whole-genome resequencing reveals signature of local adaptation and divergence in wild soybean. Evol. Appl..

[9] Zheng J., Zhao L., Zhao X., Gao T., Song N. (2022). High genetic connectivity inferred from whole-genome resequencing provides insight into the phylogeographic pattern of *Larimichthys polyactis*. Mar. Biotechnol..

[10] Saha A., Andersson A., Kurland S., Keehnen N.L., Kutschera V.E., Hössjer O., Ekman D., Karlsson S., Kardos M., Ståhl G. (2022). Whole-genome resequencing confirms reproductive isolation between sympatric demes of brown trout (*Salmo trutta*) detected with allozymes. Mol. Ecol..

[11] Zhao X., Zheng T., Gao T., Song N. (2023). Whole-genome resequencing reveals genetic diversity and selection signals in warm temperate and subtropical *Sillago sinica* populations. BMC Genom..

[12] Han Z.Q., Guo X.Y., Liu Q., Liu S.S., Zhang Z.X., Xiao S.J., Gao T.X. (2021). Whole-genome resequencing of Japanese whiting (*Sillago japonica*) provide insights into local adaptations. Zool. Res..

[13] She Z., Li L., Meng J., Jia Z., Que H., Zhang G. (2018). Population resequencing reveals candidate genes associated with salinity adaptation of the Pacific oyster *Crassostrea gigas*. Sci. Rep..

[14] Zhou C., Yang M.-j., Hu Z., Shi P., Li Y.-r., Guo Y.-j., Zhang T., Song H. (2023). Molecular evidence for the adaptive evolution in euryhaline bivalves. Mar. Environ. Res..

[15] Shan X., Hu Z., Shao C. (2020). Progress in the study of fishing-induced evolution of fish biological characteristics. Prog. Fish. Sci..

[16] Wilson A.D., Binder T.R., McGrath K.P., Cooke S.J., Godin J.-G.J. (2011). Capture technique and fish personality: Angling targets timid bluegill sunfish, *Lepomis macrochirus*. J. Fish. Res. Board Can..

[17] Peng C., Luo C., Xiang G., Huang J., Shao L., Huang H., Fan S. (2024). Genome-Wide Microsatellites in *Acanthopagrus latus*: Development, Distribution, Characterization, and Polymorphism. Animals.

[18] Lu J., Gao D., Sims Y., Fang W., Collins J., Torrance J., Lin G., Xie J., Liu J., Howe K. (2022). Chromosome-level genome assembly of *Acanthopagrus latus* provides insights into salinity stress adaptation of Sparidae. Mar. Biotechnol..

[19] Wang W., Huang J., Hu Y., Feng J., Gao D., Fang W., Xu M., Ma C., Fu Z., Chen Q. (2024). Seascapes Shaped the Local Adaptation and Population Structure of South China Coast Yellowfin Seabream (*Acanthopagrus latus*). Mar. Biotechnol..

[20] Yan C. (2012). Analysis and Evaluation of Protection and Management of the Chinese White Dolphins (*Sousa chinensis*) in Xiamen Rare Marine Species National Nature Reserve. Master’s Thesis.

[21] Yu Z., Yu J. (2017). Preliminary Study on Ecological Compensation Standard of Marine Reserve: A case of Xiamen Rare Marine Species National Nature Reserve. Mar. Environ. Sci..

[22] Zhuang Q. (2020). Study on the Gap Analysis and Establishment Management of Xiamen Marine Protected Area. Master’s Thesis.

[23] Hesp S.A. (2003). Biology of Two Species of Sparid on the West Coast of Australia. Ph.D. Thesis.

[24] Chen S., Zhou Y., Chen Y., Gu J. (2018). fastp: An ultra-fast all-in-one FASTQ preprocessor. Bioinformatics.

[25] Li H., Handsaker B., Wysoker A., Fennell T., Ruan J., Homer N., Marth G., Abecasis G., Durbin R. (2009). 1000 genome project data processing subgroup. The sequence alignment/map format and SAMtools. Bioinformatics.

[26] McKenna A., Hanna M., Banks E., Sivachenko A., Cibulskis K., Kernytsky A., Garimella K., Altshuler D., Gabriel S., Daly M. (2010). The Genome Analysis Toolkit: A MapReduce framework for analyzing next-generation DNA sequencing data. Genome Res..

[27] Layer R.M., Chiang C., Quinlan A.R., Hall I.M. (2014). LUMPY: A probabilistic framework for structural variant discovery. Genome Biol..

[28] Abyzov A., Urban A.E., Snyder M., Gerstein M. (2011). CNVnator: An approach to discover, genotype, and characterize typical and atypical CNVs from family and population genome sequencing. Genome Res..

[29] Wang K., Li M., Hakonarson H. (2010). ANNOVAR: Functional annotation of genetic variants from high-throughput sequencing data. Nucleic Acids Res..

[30] Purcell S., Neale B., Todd-Brown K., Thomas L., Ferreira M.A., Bender D., Maller J., Sklar P., De Bakker P.I., Daly M.J. (2007). PLINK: A tool set for whole-genome association and population-based linkage analyses. Am. J. Hum. Genet..

[31] Tamura K., Stecher G., Kumar S. (2021). MEGA11: Molecular evolutionary genetics analysis version 11. Mol. Biol. Evol..

[32] Alexander D.H., Novembre J., Lange K. (2009). Fast model-based estimation of ancestry in unrelated individuals. Genome Res..

[33] Durvasula A., Fulgione A., Gutaker R.M., Alacakaptan S.I., Flood P.J., Neto C., Tsuchimatsu T., Burbano H.A., Pico F.X., Alonso-Blanco C. (2017). African genomes illuminate the early history and transition to selfing in Arabidopsis thaliana. Proc. Natl. Acad. Sci. USA.

[34] Danecek P., Auton A., Abecasis G., Cornelis A.A., Banks E., DePristo M.A., Handsaker R.E., Lunter G., Marth G.T., Sherry S.T. (2011). The variant call format and VCFtools. Bioinformatics.

[35] Pickrell J., Pritchard J. (2012). Inference of population splits and mixtures from genome-wide allele frequency data. Nat. Preced..

[36] Li H., Durbin R. (2011). Inference of human population history from individual whole-genome sequences. Nature.

[37] Cantalapiedra C.P., Hernández-Plaza A., Letunic I., Bork P., Huerta-Cepas J. (2021). eggNOG-mapper v2: Functional annotation, orthology assignments, and domain prediction at the metagenomic scale. Mol. Biol. Evol..

[38] Ashburner M., Ball C.A., Blake J.A., Botstein D., Butler H., Cherry J.M., Davis A.P., Dolinski K., Dwight S.S., Eppig J.T. (2000). Gene ontology: Tool for the unification of biology. Nat. Genet..

[39] Kanehisa M., Goto S. (2000). KEGG: Kyoto encyclopedia of genes and genomes. Nucleic Acids Res..

[40] Zhu W., Xiao S., Yang C., Chen H., He T., Zeng D., Chen X., Peng M. (2021). Genetic structure analysis of *Acanthopagrus latus* populations along South China coast based on mitochondrial Cytb gene sequences. CABI Databases.

[41] Zhao H., Huang L., Zhang J., You S., Zeng Q., Liu X. (2024). Development of SNP parentage assignment techniques in the yellowfin seabream *Acanthopagrus latus*. Acta Oceanolog. Sin..

[42] Xia J.H., Huang J.H., Gong J.B., Jiang S.G. (2008). Significant population genetic structure of yellowfin seabream *Acanthopagrus latus* in China. J. Fish Biol..

[43] Sun C.-H., Gozlan R.E., Wu T., Xue D., Lao Y.-L., Yu J.-F., Zeng X.-S., Li S., Hardouin E.A., Andreou D. (2022). The role of ancestral seascape discontinuity and geographical distance in structuring rockfish populations in the Pacific Northwest. Front. Mar. Sci..

[44] Akazaki M., Tokitô A. (1982). Studies on the seedling production of yellowfin porgy (Kichinu), *Acanthopagrus latus* Houttuyn—II Egg development and metamorphoses of larvae. Aquac. Sci..

[45] Blampied S.R., Sheehan E.V., Binney F.C.T., Attrill M.J., Rees S.E. (2022). Value of coastal habitats to commercial fisheries in Jersey, English Channel, and the role of marine protected areas. Fish. Manag. Ecol..

[46] Uusi-Heikkilä S., Whiteley A.R., Kuparinen A., Matsumura S., Venturelli P.A., Wolter C., Slate J., Primmer C.R., Meinelt T., Killen S.S. (2015). The evolutionary legacy of size-selective harvesting extends from genes to populations. Evol. Appl..

[47] Li Y., Sun C., Tan Y., Li L., Zhang H., Liang Y., Zeng J., Zou H. (2020). Transcription levels and prognostic significance of the NFI family members in human cancers. PeerJ.

[48] Steele-Perkins G., Butz K.G., Lyons G.E., Zeichner-David M., Kim H.-J., Cho M.-I., Gronostajski R.M. (2003). Essential role for NFI-C/CTF transcription-replication factor in tooth root development. Mol. Cell. Biol..

[49] Cao L., Lin L., Li H., Pan X., Zhou J., Yang B., Kang Y., Qian A., Yao J., Shan X. (2022). Role of Ras-related C3 botulinum toxin substrate (Rac2) in northern snakehead (*Channa argus*) resistance to Aeromonas veronii infection. Aquacult. Res..

[50] Laing K.J., Purcell M.K., Winton J.R., Hansen J.D. (2008). A genomic view of the NOD-like receptor family in teleost fish: Identification of a novel NLR subfamily in zebrafish. BMC Evol. Biol..

[51] Lessieur E.M., Joseph F., Gaivin R.J., Ping S., Perkins B.D. (2017). The Ciliopathy Gene ahi1 Is Required for Zebrafish Cone Photoreceptor Outer Segment Morphogenesis and Survival. Investig. Ophthalmol. Visual Sci..

[52] Yang H., Liu C., Jamsen J., Wu Z., Wang Y., Chen J., Zheng L., Shen B. (2012). The DNase domain-containing protein TATDN1 plays an important role in chromosomal segregation and cell cycle progression during zebrafish eye development. Cell Cycle.

[53] Araya N., Arimura H., Kawahara K.-I., Yagishita N., Ishida J., Fujii R., Aratani S., Fujita H., Sato T., Yamano Y. (2009). Role of Kenae/CCDC125 in cell motility through the deregulation of RhoGTPase. Int. J. Mol. Med..

[54] Wang X., Li T., Sukrithan V., Ratan A., McCarter M., Carpten J.D., Colman H., Ikeguchi A., Puzanov I., Arnold S.M. (2023). Incorporating long non-coding RNA (lncRNA) into genome-wide biomarker screening for prognostic gene signatures of immunotherapy outcomes. J. Clin. Oncol..

[55] Zhang B., Li J., Ma Y., Dong F., Tao Z., Shen J., Tian Y., Shi F., Lu L. (2010). Molecular cloning and sequence analysis of duck BLVRA gene. CABI Databases.

[56] Johnson A.C., Wu W., Showmaker K.C., Lindsey M.L., Garrett M.R. (2019). Physiological Omics Identifies Mechanisms that Attenuate Renal Injury and Blood Pressure in Dahl salt-sensitive Arhgef11−/− Rats. FASEB J..

[57] Tu J., Huang C., Huang Y., Wang H. (2020). Molecular cloning and expression analysis of jphs in *Megalobrama amblycephala*. J. Huazhong Agric. Univ..

